# Striatal phosphodiesterase 10A availability is altered secondary to chronic changes in dopamine neurotransmission

**DOI:** 10.1186/s41181-016-0005-5

**Published:** 2016-03-21

**Authors:** Maarten Ooms, Sofie Celen, Ronald De Hoogt, Ilse Lenaerts, Johnny Liebregts, Greet Vanhoof, Xavier Langlois, Andrey Postnov, Michel Koole, Alfons Verbruggen, Koen Van Laere, Guy Bormans

**Affiliations:** 1 Department of Pharmaceutical and Pharmacological Sciences KU Leuven, Laboratory for Radiopharmacy, Campus Gasthuisberg O&N 2, Herestraat 49 bus 821, 3000 Leuven, Belgium; 2 Janssen Research & Development, a Division of Janssen Pharmaceutica NV, Beerse, Belgium; 3 Division of Nuclear Medicine, KU Leuven and University Hospital Leuven, Leuven, Belgium

**Keywords:** Phosphodiesterase 10A, Dopamine neurotransmission, D-amphetamine, Small animal PET, Brain imaging

## Abstract

**Background:**

Phosphodiesterase 10A (PDE10A) is an important regulator of nigrostriatal dopamine (DA) neurotransmission. However, little is known on the effect of alterations in DA neurotransmission on PDE10A availability. Here, we used [^18^F]JNJ42259152 PET to measure changes in PDE10A availability, secondary to pharmacological alterations in DA release and to investigate whether these are D_1_- or D_2_-receptor driven.

**Results:**

Acute treatment of rats using D-amphetamine (5 mg, s.c. and 1 mg/kg i.v.) did not result in a significant change in PDE10A BP_ND_ compared to baseline conditions. 5-day D-amphetamine treatment (5 mg/kg, s.c.) increased striatal PDE10A BP_ND_ compared to the baseline (+24 %, *p* = 0.03). Treatment with the selective D2 antagonist SCH23390 (1 mg/kg) and D-amphetamine decreased PDE10A binding (-22 %, *p* = 0.03). Treatment with only SCH23390 further decreased PDE10A binding (-26 %, *p* = 0.03). No significant alterations in PDE10A mRNA levels were observed.

**Conclusions:**

Repeated D-amphetamine treatment significantly increased PDE10A binding, which is not observed upon selective D_1_ receptor blocking. This study suggests a potential pharmacological interaction between PDE10A enzymes and drugs modifying DA neurotransmission. Therefore, PDE10A binding in patients with neuropsychiatric disorders might be modulated by chronic DA-related treatment.

**Electronic supplementary material:**

The online version of this article (doi:10.1186/s41181-016-0005-5) contains supplementary material, which is available to authorized users.

## Background

Dopamine (DA) is an important neurotransmitter in the brain and in particular in the striatum, which is the primary input of the basal ganglia. DA neurotransmission plays an important role in the regulation of motor, reward and cognitive processes. Alterations in DA neurotransmission are key hallmarks in the pathogenesis of several diseases in these domains such as Huntington’s disease, Parkinson’s disease, addiction and schizophrenia (Schmidt and Reith 2010).

Medium spiny neurons (MSNs) represent 90–95 % of the neurons in striatum. MSNs are GABAergic projection neurons that integrate dopaminergic and glutamatergic neurotransmission. Two major pathways of MSNs are known. MSNs of the direct pathway are striatonigral neurons that project to the internal part of the globus pallidus and the substantia nigra pars reticularis. Activation of the direct pathway inhibits the GABAergic output of these two nuclei on the thalamus and thereby stimulates behavioral activity. The indirect pathway consists of striatopallidal MSNs that project to the external globus pallidus. Activation of the indirect pathway removes the inhibition of the external globus pallidus on the subthalamic nucleus. This ultimately increases the inhibition of the globus pallidus and substantia nigra on the thalamus and inhibits behavioral activity (Albin et al. 1989).

In the MSNs, two main DA receptor pathways can be distinguished. Both the D_1_ receptor and the D_2_ receptor mediate their effects through activation or inactivation of the 3’,5’-cyclic adenosine monophosphate/Protein Kinase A (cAMP/PKA) pathway (Nishi et al. 2008). Although all MSNs are sensitive to DA neurotransmission, a distinct distribution pattern of the DA receptor subtypes exists. Striatonigral MSNs of the direct pathway predominantly express the D_1_ receptor. When stimulated, D_1_ receptors activate adenylyl cyclase (AC) which initiates the cAMP/PKA cascade. Striatopallidal neurons on the other hand predominantly express D_2_ receptors. Stimulation of these G_i_ mediated receptors inhibits AC resulting in an inactivation of the indirect pathway (Fisone et al. 2007; Traynor and Neubig 2005).

Since striatal excitability depends on activation or inactivation of the cAMP/PKA pathway, concentrations of cAMP play a central role in the regulation of the MSNs. The intracellular concentration of cAMP is determined by the balance between its production and its degradation. Production of cAMP in striatum is controlled by activation of AC while the degradation of cAMP is catalyzed by a class of enzymes called phosphodiesterases (PDEs).

PDE10A is a subfamily of PDEs which can hydrolyze both cAMP and cGMP, although its affinity for cAMP is higher (Fujishige et al. 1999). It has a very limited distribution and is mainly expressed in the MSNs of the striatum and substantia nigra (Lakics et al. 2010; Seeger et al. 2003). The role of PDE10A in cAMP metabolism, together with its unique localization makes PDE10A a principal regulator of nigrostriatal DA neurotransmission.

Several research groups have focused on the relationship between PDE10A activity and cAMP levels. Research conducted by Jäger et al. suggested that PDE10A is activated by high concentrations of cAMP (Jäger et al. 2012). Since changes in DA neurotransmission directly influence cAMP levels in striatum, it is plausible that changes in DA neurotransmission might also induce changes in PDE10A activity. Although several authors have investigated how PDE10A inhibition may modify DA neurotransmission (Nishi et al. 2008; Sotty et al. 2009; Gresack et al. 2013), to our knowledge, few studies have examined the modulation of PDE10A expression and activity by DA neurotransmission.

Dlaboga et al were the first to investigate the effect a chronic treatment with a D_2_ antagonist on PDE10A expression levels (Dlaboga et al. 2008). They noticed decreased PDE10A expression after chronic treatment with haloperidol. These data were later contradicted by Natesan et al., who could not detect any change in PDE10A expression and PET binding after chronic haloperidol treatment (Natesan et al. 2014). Finally, an attempt to understand the relationship between DA neurontransmission and PDE10A was done by Giorgi et al. (Giorgi et al. 2011). They noticed that loss of dopaminergic nigrostriatal neurons significantly decreased PDE10A mRNA levels in striatum.

These studies suggest that changes in DA neurotransmission might induce changes in cAMP levels and PDE10A expression. This is however still controversial. In this study we aimed to evaluate changes in PDE10A activation or expression secondary to changes in DA neurotransmission. In order to test this hypothesis, we used positron emission tomography (PET) with [^18^F]JNJ42259152, a validated PET tracer for PDE10A (Celen et al. 2013; Van Laere et al. 2013a, b; Andrés et al. 2011), to quantify in vivo alterations in PDE10A binding secondary to stimulation of the DA system. Since D_1_ and D_2_ receptors influence cAMP levels in opposite ways, we evaluated the relative importance of D_1_ and D_2_ receptors in such response to DA alterations, using various pharmacological treatment schedules and imaging with the D_2_ antagonist [^11^C]raclopride.

## Methods

### Animals

Healthy female Wistar rats (body weight of 200–250 g at start of experiments) were used. The animals were housed in individually ventilated cages in a thermoregulated (22 °C) and humidity-controlled environment under a 12 h/12 h day/night cycle with free access to food and water. All animal experiments were conducted according to the Belgian code of practice for the care and use of animals and with approval of the KU Leuven ethical committee for animal experiments.

### Animal treatment

Rats were divided into eight different treatment groups which are outlined in Table 1.

**Table 1:** Overview of the different treatment groups (* saline treatment, equivalent volumes)

Experiment	Treatment	Injection route	Dose (mg/kg)	Duration of treatment	Time before scan	n
1	Amphetamine	SC	5	Acute	60 min	6
2	Amphetamine	IV	1	Acute	5 min	5
3	Saline (test/retest)	SC	*	Acute	60 min	3
4	Saline (test/retest)	IV	*	Acute	5 min	3
5	Amphetamine	SC	5	5 days	4 h	5
6	Amphetamine + SCH23390	SC	5 1	5 days	4 h	6
7	SCH23390	SC	1	5 days	4 h	5
8	Saline	SC	*	5 days	4 h	3

Rats undergoing acute treatment were scanned at baseline conditions and after acute treatment. The 5 day treatment consisted of a baseline scan, a scan immediately after the 5 day treatment and a scan 10 days after the last treatment

#### Single-dose treatment

At the start of the experiment, animals were scanned using [^18^F]JNJ42259152 prior to any treatment to asses baseline BP_ND_ values. In order to evaluate the acute, single-dose effects of D-amphetamine administration on PDE10A, rats were treated with D-amphetamine (5 mg/kg, solution 2.5 mg/ml in saline, s.c. in awake animals) and were scanned with [^18^F]JNJ42259152 60 min after D-amphetamine injection (Experiment 1; *n* = 6). A second group of rats (Experiment 2; *n* = 5) was scanned 5 min after D-amphetamine treatment (1 mg/kg, i.v. in anaesthetized rats). Striatum BP_ND_ values for [^18^F]JNJ42259152 after treatment were compared to baseline BP_ND_ values acquired in the same rat. A similar treatment protocol with saline (2 ml/kg, s.c.) was used as a control to check for the potential effect of stress induced by the acute treatment protocols. Additionally, scans in saline treated animals were also used to calculate the test-retest variability of basal BP_ND_ values as a function of time (Experiments 3 and 4; *n* = 3 per group). Test-retest variability was calculated as |BP_1_ – BP_2_| / (BP_1_ + BP_2_) × 200 with BP_1_ and BP_2_ the BP_ND_ values from the baseline and saline treated scan respectively acquired in the same animal. For all experiments, there was a maximum time-gap of 1 week between baseline scan and test scan.

#### Chronic treatment

The effect of chronic dosage with D-amphetamine was evaluated after a 5-day schedule. In order to evaluate the relative contribution of D_1_ versus D_2_ receptors in the D-amphetamine response, rats were also treated with SCH23390, a known D_1_ receptor antagonist (Bourne 2001). All rats were first scanned at baseline conditions prior to any treatment using [^18^F]JNJ42259152. After their baseline scans, the different groups of animals were subjected to different treatment protocols (See Table 1). The treatment protocol consisted of subcutaneous injection in awake animals for five consecutive days with 5 mg/kg D-amphetamine (Experiment 5; *n* = 5), a combination of 5 mg/kg D-amphetamine and 1 mg/kg SCH23390 (Experiment 6; *n* = 6) or 1 mg/kg SCH23390 alone (Experiment 7; *n* = 5) once per day. At the fifth day of treatment, rats were scanned using [^18^F]JNJ42259152. A time gap of 4 h was left between the final injection and the start of the microPET scan in order to exclude any potential acute effects of the treatment. Finally, the rats were left untreated for another ten days after which the PDE10A binding potentials were again determined. A subset of the animals treated with only D-amphetamine (Experiment 5; *n* = 3) was additionally scanned with [^11^C]raclopride to visualize D_2_-receptor availability at the same time points. The influence of the chronic D-amphetamine treatment on PDE10A BP_ND_ values was assessed in a control group of rats treated for 5 consecutive days with equivalent volumes (2 ml/kg) of saline (Experiment 8; *n* = 3).

### Radiochemistry

[^18^F]JNJ42259152 was radiolabeled by alkylation of the corresponding precursor with [^18^F]fluoroethyl bromide following a previously published method (Andrés et al. 2011). The radiotracer was obtained with a radiochemical purity > 98 % and a specific activity of 62–193 GBq/μmol (injected mass dose = 0.3–9 μg/kg) at the time of injection. [^11^C]Raclopride was synthesized according to a previously published method (Van Laere et al. 2010) with a radiochemical purity >98 % and a specific activity of 103–120 GBq/μmol (injected mass dose = 0.3–1.1 μg/kg) at time of injection.

### Small animal PET imaging

Small animal PET imaging was performed with a FOCUS 220 tomograph (Siemens/Concorde Microsystems, Knoxville, TN). Rats were anesthetized and kept under anesthesia during the entire scan using 2.5 % isoflurane in oxygen (1 L/min). Animals were injected intravenously with about 50 MBq of [^18^F]JNJ42259152 or about 70 MBq of [^11^C]raclopride and scanned dynamically for 90 min. Data were acquired in a 128x128x95 matrix with a pixel width of 0.949 mm and a slice thickness of 0.796 mm. The scan data were acquired in list mode. Acquisition data were Fourier re-binned in 24 time frames (4 × 15 s, 4 × 60 s, 5 × 180 s, 8 × 5 min, 3 × 10 min) and reconstructed using maximum a posteriori iterative reconstruction (MAP; 18 iterations, 9 subsets, fixed resolution: 1.5 mm). The summed images (all timeframes) of the reconstructed data were spatially normalized to an in-house created [^11^C]raclopride template of the Wistar rat brain. The affine transformation was then used to normalize all time frames of the dynamic small animal PET data set to allow automated and symmetric volumes of interest (VOIs) analyses. Time activity curves (TACs) were generated for striatum and cerebellum using PMOD software (v 3.2, PMOD Technologies, Zurich, Switzerland). For [^18^F]JNJ42259152, striatal binding potential values (BP_ND_) in striatum were extracted from the TACs using a Logan reference plot as previously validated by Celen and coworkers (Celen et al. 2013). K2’ and t* values for the Logan reference analysis were estimated using a two-tissue reference model. [^11^C]Raclopride binding was quantified using BP_ND_ values derived from a simplified reference tissue model as previously described (Ikoma et al. 2009). Cerebellum was used as a reference region for both reference tissue models to quantify [^18^F]JNJ42259152 and [^11^C]raclopride binding in different brain regions. PDE10A BP_ND_ images were generated by voxel based parametric mapping. Voxelwise parametric BP_ND_ images were constructed using a Logan reference tissue model using the cerebellum as reference region. For [^11^C]Raclopride, BP_ND_ images were generated using a SRTM approach.

### Quantitative analysis of striatal mRNA levels

#### Tissue extraction

Healthy female Wistar rats were treated for five consecutive days with D-amphetamine (*n* = 9, 5 mg/kg, s.c.) or equivalent volumes of saline (*n* = 9). At the final day of treatment, rats were anaesthetized using 2.5 % isoflurane in oxygen (1 L/min) and sacrificed by decapitation. Striatum was then isolated and used for mRNA quantification.

#### Real-time quantitative PCR

RNA purification from striatum was performed with an RNeasy kit (Qiagen, Hilden, Germany), including DNaseI digestion, and extracted RNA was eluted with RNase-free H_2_O. 1.2 μg RNA was used for subsequent cDNA synthesis using random primers and SuperScript® III First-Strand Synthesis System (Invitrogen, Carlsbad, US) according to manufacturer’s protocol.

Real-Time Quantitative PCR (RTQ-PCR) was performed on an ABI Prism 7900-HT Sequence Detection System (Applied Biosystems, Lennik, Belgium). A qPCR core kit without dUTP (Eurogentec, Seraing, Belgium) was combined, according to protocol, with pre-designed Taqman Gene Expression Assays (Applied Biosystems) to quantify the genes of interest; PDE10A (Rn00673152_m1), D_1_ receptor (Rn03062203_s1) and D_2_ receptor (Rn00561126_m1) and internal control genes corresponding to glucuronidase b (GUSB, Rn00566655_m1), hydroxymethylbilane synthase (HMBS, Rn00565886_m1), phosphoglycerate kinase 1 (PGK1, Rn00821429_g1), peptidylpropyl isomerase b (PPIB, Rn03302274_m1) and transferrin receptor (TFRC, Rn01474701_m1, all Applied Biosystems). Serial dilutions of cDNA were used to generate standard curves with all Taqman assays in order to calculate PCR efficiencies (all between 95 % and 105 %) and quantify expression levels. Samples were assessed in duplicate. Finally GeNorm software was used to identify the most stably expressed internal control genes (http://genomebiology.com/2002/3/7/research/0034). These genes were then used to normalize PDE10A, D_1_ and D_2_ receptor expression.

### General statistics

Reported values are reported as mean ± SD. Conventional statistical analysis was carried out using Graphpad Prism 5.1 (Graphpad Software, La Jolla, CA, US). A non-parametric Wilcoxon signed rank test was performed to compare BP_ND_ values at the different time points in each treatment group. Significance was accepted at the 95 % probability level.

## Results

### Saline treated animals

In order to test the effect of treatment protocols on PDE10A BP_ND_ values, rats were treated with equivalent volumes of saline (Experiments 3, 4 and 8). When comparing PDE10A BP_ND_ values after acute saline treatment (average BP_ND_ = 1.40 ± 0.20) and at baseline conditions (BP_ND_ = 1.61 ± 0.11), no significant changes could be found (Table 2). Additionally, the same BP_ND_ values derived from the acute saline treatment rats were used to calculate test-retest variability as described earlier. Individual and average test-retest variability are shown in Table 2. Likewise, in the chronically saline-treated animals (Experiment 8) no significant changes could be detected (BP_ND_ = 2.61 ± 0.45; 2.51 ± 0.13; 2.27 ± 0.61 at baseline, day 5 and day 15 of the experiment respectively).

**Table 2:** Test-retest statistics for [^18^F]JNJ42259152 in rat striatum

Animal	BP1 (baseline)	BP2 (saline treated)	Test-retest variability (%)
1	1.71	1.21	34.8
2	1.48	1.53	2.5
3	1.58	1.22	25.9
4	1.51	1.69	10.4
5	1.62	1.24	26.3
6	1.69	1.51	11.9
Mean ± SD	1,60 ± 0.09	1.40 ± 0.20	18.6 ± 12.2

Test retest variability was calculated as |BP_1_ – BP_2_| / (BP_1_ + BP_2_) × 200

### Single dose D-amphetamine treatment

**Fig. 1:**
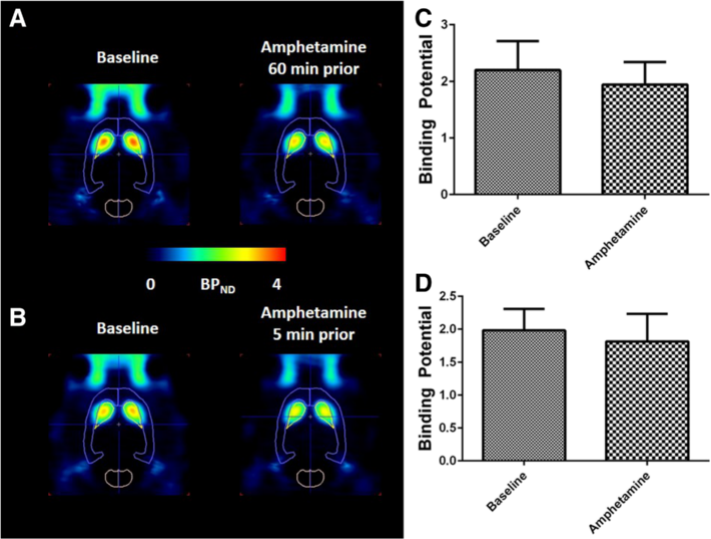
[^18^F]JNJ42259152 BP_ND_ values after acute amphetamine treatment. *Left*: Transversal images of PDE10A binding in the brain at baseline conditions vs 60 min after s.c. amphetamine treatment (**a**) and at baseline conditions vs 5 min after i.v. amphetamine treatment (**b**). Images are overlaid on a VOI map and presented as averaged, parametric BP_ND_ images (Logan Reference) at baseline conditions and amphetamine treated conditions. *Right*: Comparison of BP_ND_ in striatum at baseline conditions and 1 h after subcutaneous injection of amphetamine (**c**) and 5 min after intravenous injection of amphetamine (**d**). No significant difference was found

Representative images of PDE10A BP_ND_ values for [^18^F]JNJ42259152 in the brain acquired at baseline conditions and after single dose treatment with D-amphetamine are presented in Fig. 1a-b. Single dose treatment with D-amphetamine did not influence striatal PDE10A binding. Quantification of BP_ND_ values in experiment 1 showed no change in PDE10A BP_ND_ between baseline conditions (BP_ND_ = 2.20 ± 0.51) and 60 min after subcutaneous administration of D-amphetamine (BP_ND_ = 1.95 ± 0.39, Fig. 1c)). Similarly, the rats of experiment 2 did not have significantly different BP_ND_ 5 min after i.v. D-amphetamine injection (BP_ND_ = 1.82 ± 0.42) compared to baseline conditions (BP_ND_ = 1.98 ± 0.33; Fig. 1d).

### Chronic treatment

#### Five day D-amphetamine treatment (Experiment 5)

**Fig. 2:**
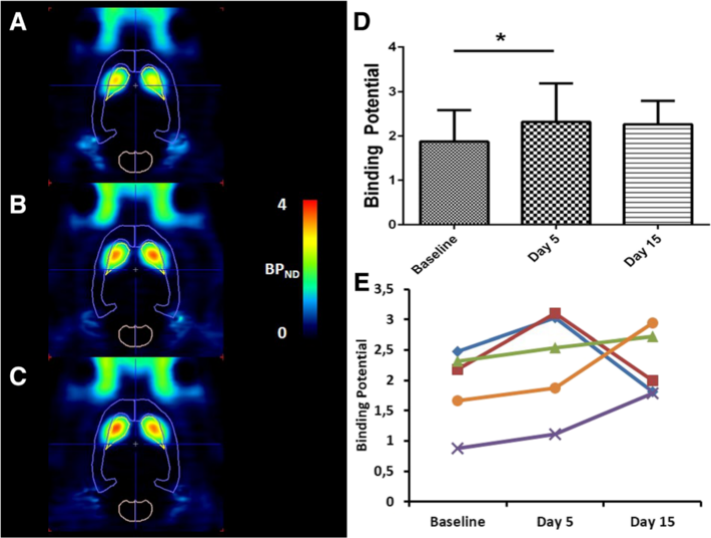
[^18^F]JNJ42259152 BP_ND_ values after a 5-days amphetamine treatment. *Left*: Transversal images of PDE10A binding in brain. Images are overlaid on a VOI map and presented as averaged, parametric BP_ND_ images (Logan Reference) at baseline conditions (**a**), at day 5 (**b**) and day 15 (**c**) of the experiment. *Right*: Averaged (**d**) and individual (**e**) BP_ND_ values in striatum of amphetamine treated rats at baseline conditions, after 5 days of amphetamine treatment (day 5) and after 10 days of washout (day 15). Each line represents a repeated measurement in a single rat at different stages of treatment. Averaged data are presented as mean ± SD. Wilcoxon matched pair test; *p < 0.05

After treatment with D-amphetamine for five consecutive days, striatal PDE10A BP_ND_ was 24 ± 12 % higher (BP_ND_ = 2.28 ± 0.76) compared to the baseline values (BP_ND_ = 1.84 ± 0.60; baseline vs day 5: *p* = 0.03, Fig. 2). After a washout period of 10 days, average BP_ND_ values were slightly higher (BP_ND_ = 2.25 ± 0.54) compared to baseline conditions, however this difference was not significant (baseline vs day 15; *p* = 0.22).

**Fig. 3:**
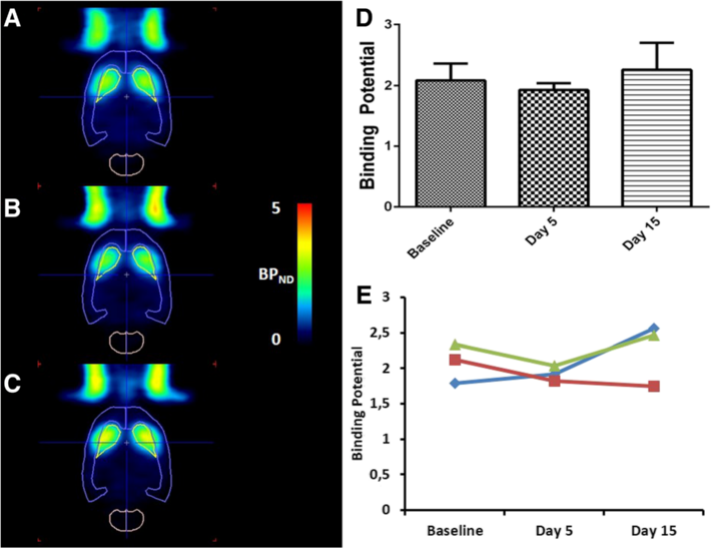
[^11^C]Raclopride BP_ND_ values after a 5-days amphetamine treatment. *Left*: Transversal images of D_2_ receptor binding in the brain. Images are overlaid on a VOI map and presented as averaged, parametric BP_ND_ images (SRTM) at baseline conditions (**a**), at day 5 (**b**) and day 15 (**c**) of the experiment. *Right*: Averaged (**d**) and individual (**e**) BP_ND_ values in striatum of amphetamine treated rats at baseline conditions, after 5 days of amphetamine treatment (day 5) and after 10 days of washout (day 15). Each line represents a repeated measurement in a single rat at different stages of treatment. Averaged data are presented as mean ± SD. No significant difference was observed

On a subset of these animals (*n* = 3), D_2_ receptor availability was quantified using [^11^C]raclopride. No significant difference in striatal D_2_ receptor binding could be found between the baseline conditions (BP_ND_ = 2.08 ± 0.28) and day 5 (BP_ND_ = 1.93 ± 0.11) or day 15 (BP_ND_ = 2.25 ± 0.45) of the experiment (Fig. 3) (baseline vs day 5, *p* = 0.25; baseline vs day 15, *p* = 0.37).

#### Five day SCH23390 and D-amphetamine treatment (Experiment 6)

**Fig. 4:**
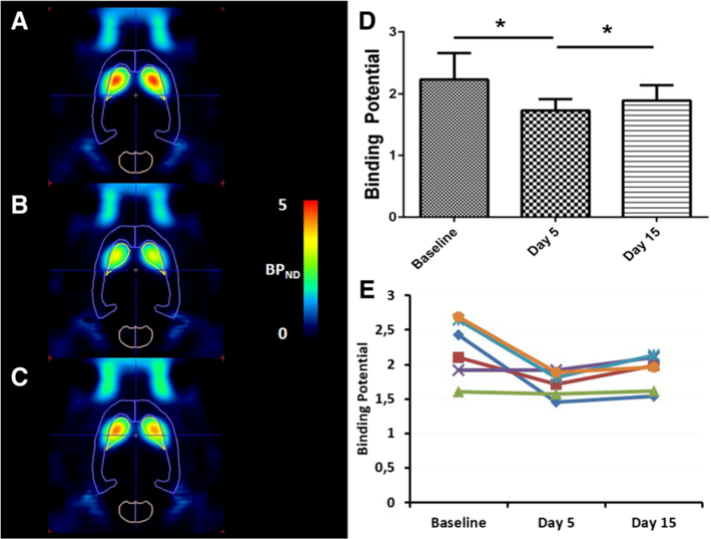
[^18^F]JNJ42259152 BP_ND_ values after 5 days treatment with amphetamine and SCH23390. *Left*: Transversal images of PDE10A binding in the brain. Images are overlaid on a VOI map and presented as averaged, parametric BP_ND_ images (Logan Reference) at baseline conditions (**a**), at day 5 (**b**) and day 15 (**c**) of the experiment. *Right*: Averaged (**d**) and individual (**e**) BP_ND_ values in striatum of SCH23390 + amphetamine treated rats at baseline conditions, after 5 days of amphetamine treatment (day 5) and after 10 days of washout (day 15). Each line represents a repeated measurement in a single rat at different stages of treatment. Averaged data are presented as mean ± SD. Wilcoxon matched pair test; *p < 0.05

Treatment of rats with a combination of the D_1_ receptor antagonist SCH23390 and D-amphetamine decreased PDE10A binding (Experiment 6, Fig. 4). Binding potentials were 20 ± 16 % lower after five consecutive days of treatment (BP_ND_ = 1.73 ± 0.18) compared to baseline conditions (BP_ND_ = 2.23 ± 0.43) acquired in the same animals (baseline vs day 5; *p* = 0.03). After an additional 10-days washout period, PDE10A binding potentials augmented back to PDE10A binding at baseline conditions (BP_ND_ = 1.89 ± 0.25; baseline vs day 15 *p* = 0.11).

#### Five day SCH23390 treatment (Experiment 7)

**Fig. 5:**
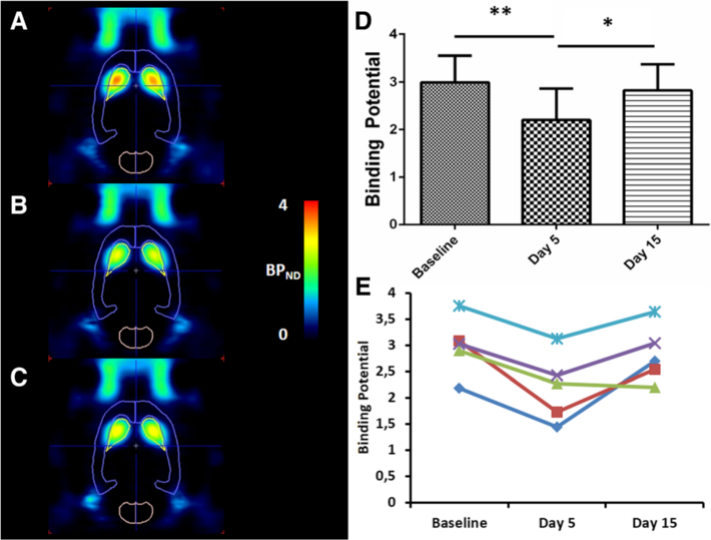
[^18^F]JNJ42259152 BP_ND_ values after 5 days treatment with SCH23390. *Left*: Transversal images of PDE10A binding in the brain. Images are overlaid on a VOI map and presented as averaged, parametric BP_ND_ images (Logan Reference) at baseline conditions (**a**), at day 5 (**b**) and day 15 (**c**) of the experiment. *Right*: Averaged (**d**) and individual (**e**) BP_ND_ values in striatum of SCH23390 treated rats at baseline conditions, after 5 days of amphetamine treatment (day 5) and after 10 days of washout (day 15). Each line represents a repeated measurement in a single rat at different stages of treatment. Wilcoxon matched pair test; *p < 0.05, **p < 0.005

In order to study the BP_ND_ changes due to SCH23390 treatment in the absence of D-amphetamine, another group of rats (Experiment 7, Fig. 5) was treated for five consecutive days with the D_1_ receptor antagonist only. Also in this case treatment resulted in a significant decrease of PDE10A binding (BP_ND_ = 2.20 ± 0.52) compared to baseline conditions (BP_ND_ = 2.99 ± 0.59, *p* = 0.03). This decrease was however slightly higher compared to the decrease observed after treatment with a combination of D-amphetamine and SCH32290 (- 22 % after SCH23390 and D-amphetamine treatment versus – 27 ± 10 % after SCH23390 treatment). After a washout period of 10 days, binding potentials returned to the levels of the baseline scan (BP_ND_ = 2.82 ± 0.55, Fig. 5; baseline vs day 15: *p* = 0.22).

### Quantitative analysis of striatal mRNA levels

**Fig. 6:**
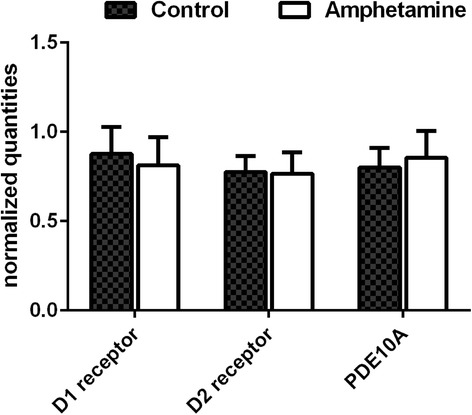
mRNA quantification in amphetamine and saline treated animals. Normalized quantities for D_1_, D_2_ and PDE10A acquired in amphetamine and saline treated. GUSB and HMBS were used for the normalization of quantities values. Data are presented as mean ± SD. No significant difference in mRNA levels of amphetamine and saline treated animals could be found

Identification of the most stable household gene using GeNorm demonstrated that GUSB and HMBS were the most stable. These two genes where therefore used for normalization of D_1_ receptor, D_2_ receptor and PDE10A expression. Normalized C_T_ values for D_1_, D_2_ and PDE10A acquired in D-amphetamine and saline treated rats are displayed in Fig. 6. After comparison of normalized D_1_, D_2_ and PDE10A mRNA levels in stratum of saline and D-amphetamine treated animals, no significant alterations could be detected.

## Discussion

Alterations in DA neurotransmission are key pathological hallmarks in many neurological and psychiatric disorders such as Huntington’s disease, Parkinson’s disease, addiction and schizophrenia (Schmidt and Reith 2010). Furthermore, altering DA neurotransmission is an important mechanism of several drugs commonly used in the treatment of these diseases. Since PDE10A is dominantly expressed in the striatal MSNs, we hypothesized that alterations in DA neurotransmission might also influence PDE10A binding through feedback on the cAMP/PKA pathway.

In the present study, we used [^18^F]JNJ42259152 small animal PET to quantify PDE10A binding in vivo. The quantification of PDE10A using BP_ND_ values acquired by Logan reference plot has been extensively evaluated by Celen et al. (Celen et al. 2013). When comparing different BP_ND_ values at baseline conditions acquired over the different experiments, a broad range of baseline BP_ND_ values in the different experiments was observed. The reason of this variation was not clear. JNJ42259152 is a PDE10A inhibitor, so potentially a mass dose effect need to be taken into consideration. The specific activity range of [^18^F]JNJ42259152 at the time of injection showed a relatively broad range (62–196 GBq/μmol, administered mass dose 0.14–0.45 μg) that originates from the fact that multiple scan sessions were conducted with the same batch of [^18^F]JNJ42259152. For each individual rat however, the specific activity at the start of the scan did not differ much between the scans obtained at different time points. In addition, we did not observe any correlation between BP_ND_ values and specific activity of the tracer (Additional file 1). Additionally, since anesthesia might have an influence on the response to D-amphetamine (McCormick et al. 2011), the level of isoflurane used to anesthetize the animals was kept constant throughout the whole experiment.

Therefore, we can assume that the large variation of baseline BP_ND_ values is probably a result of inter-individual variation such as deviations in baseline PDE10A expression levels or PDE10A activity. To investigate this hypothesis, we compared BP_ND_ values acquired in rats from the different provides in this study (Harlan and Janvier). Our data suggested that the baseline BP_ND_ values acquired in Janvier Wistar rats were significantly higher compared to the BP_ND_ values acquired in Haran Wistar rats (*p* = 0.001; Additional file 2). Therefore we can conclude that there could indeed be a large inter-individual variation causing the broad range of baseline BP_ND_ values.

One of the main advantages of PET imaging however is that it allows to repeatedly scan the same animal under different conditions, provided a suitable reference region can be found. Due to the selective expression of PDE10A in striatum, cerebellum can be used as a reference region for PDE10A quantification using [^18^F]JNJ42259152 (Celen et al. 2013; Van Laere et al. 2013b). Hence, the baseline scan of an animal can be used as an internal control for the PET scans obtained in several conditions in the same animal. This allows paired statistical analysis which looks at changes in BP_ND_ values during the different treatment stages rather than comparing absolute group-averaged BP_ND_ values. Therefore, the bias caused by inter-individual variation on the results is expected to be minimal. Additionally, we determined the test-retest variability of the saline treated animals. In the different experiments, the changes in PDE10A BP_ND_ values are larger than the calculated test-retest variability. Therefore we can conclude that the differences observed in our study are significantly larger than the statistical variation in baseline PDE10A BP_ND_ levels.

The treatment protocols in this study were designed to evaluate the effects of alterations in DA neurotransmission on striatal PDE10A binding. In order to stimulate DA release, we treated animals with 5 mg/kg D-amphetamine (s.c.), a dose commonly used in literature for D-amphetamine treatment (Yin et al. 2006; Shi and McGinty 2011). Injection of animals with D-amphetamine increases synaptic DA levels by blocking and reversing the DA reuptake transporter (Robertson et al. 2009). Striatal DA release secondary to amphetamine was quantified by Kuczenski et al (Kuczenski 1986). Their research showed that at a dose of 3 mg/kg amphetamine, a maximum DA release in striatum was achieved which could not be increased by using higher dosages. Therefore, a maximum effect can be expected after amphetamine dosage of 5 mg/kg. In striatum, the increased DA release can potentially stimulate both the D_1_ as the D_2_ receptor pathway resulting in increased (D_1_) or decreased (D_2_) cAMP levels in the postsynaptic neurons (Albin et al. 1989). Since the expression of PDE10A is also most pronounced in these neurons (Lakics et al. 2010; Seeger et al. 2003), it is likely that the alterations in cAMP might also induce changes in PDE10A activity and/or expression as an effective compensating mechanism to normalize cAMP levels.

Recent research showed that the effect of PDE10A inhibitors was dependent on the activation state of both the direct as the indirect pathway of DA neurotransmission (Megens et al. 2014). Further evidence on the differential effect of PDE10 inhibition depending on the activation state of D_1_ and D_2_ pathways can be found in the behavioral differences in different PDE10A KO mice. C57 KO mice with genetic background of C57Bl6 (high dopaminergic tone) show increase in amphetamine-stimulated locomotor activity (Siuciak et al. 2008), while PDE10 DBA KO mice have similar amphetamine-stimulated locomotor activity as WT mice (Siuciak et al. 2006). These data indeed suggest that there is an interaction between PDE10A and DA neurotransmission. Additionally these data could indicate that both the D_1_ as the D_2_ pathway influence PDE10A in different ways.

Since the stress of the treatment protocols can induce several physiological changes, the treatment itself might also influence PDE10A microPET acquisition and/or PDE10A expression. In order to evaluate the influence of the treatment protocol, treatment with saline was included as control (Experiments 3, 4 and 8). Both acute and chronic treatment with saline did not result in a change of PDE10A BP_ND_ values, indicating that the treatment protocol as such did not affect the results to a measurable extent.

In the first two experiments we tested the effect of acute D-amphetamine treatment on PDE10A tracer binding. PDE10A binding after acute D-amphetamine treatment did not change significantly compared to baseline conditions. As stated above, postsynaptic neurons might adapt to changes in cAMP by modifying hydrolysis of cAMP. In literature, several lines of evidence imply increased cAMP levels in striatum secondary to D-amphetamine treatment (Ren et al. 2009; Simpson et al. 1995). Ren et al. investigated cAMP levels in different brain regions 10 min after an i.v. challenge of amphetamine (doses: 0.25–3 mg/kg). They demonstrated 50 % higher cAMP levels after i.v. injection of amphetamine (>1 mg/kg) compared to baseline. The lack of PDE10A binding change observed in our initial data may be explained by the short time period between D-amphetamine treatment and the PDE10A microPET determination. Jaber et al. reported no change in D_1_ or D_2_ receptor transcription three hours after injection of 5 mg/kg D-amphetamine (Jaber et al. 1995). This lack of alteration in the DA receptors could indicate that the expression of a more downstream effector of the DA neurotransmitter such as PDE10A might not be changed either.

Additionally, literature data has shown that the D_1_ and D_2_ receptors affect AC activity (and as a result also the cAMP levels) in opposing ways (Albin et al. 1989). Since changes in cAMP would be the driving force for changes in PDE10A (Jäger et al. 2012), both receptors would also induce opposite changes in PDE10A. To our knowledge, there is no evidence of a differential selectivity of DA between the D_1_ receptor pathway and the D_2_ receptor pathway when animals are treated with D-amphetamine at dosages similar to the dose used in our experiment. This could mean that despite the potential local change in cAMP levels after acute D-amphetamine injection, it is plausible that the overall striatal cAMP concentrations remain unchanged. As a result, the average PDE10A levels in all the neurons of striatum would also remain unchanged. Furthermore, desensitization of the D_1_ receptor pathway (Roseboom and Gnegy 1989) and upregulation of the presynaptic D_2_ receptor (Tomić et al. 1997) were observed secondary to acute D-amphetamine treatment. This implies that although cAMP levels rapidly increase after acute D-amphetamine treatment (Ren et al. 2009; Simpson et al. 1995), compensating mechanisms, such as receptor internalization (Skinbjerg et al. 2010) or desensitization (Roseboom and Gnegy 1989) can normalize cAMP levels and minimize the impact on downstream effectors such as PDE10A.

In chronic D-amphetamine treatment on the other hand, it is generally accepted that the D_1_ receptor plays a more dominant role in the development of sensitization (Shi and McGinty 2011; Wright et al. 2013; Vanderschuren and Kalivas 2000). After 5 days of D-amphetamine treatment, PDE10A binding was found to be significantly higher compared to baseline conditions. The increase in PDE10A binding could conceivably be an indirect and compensating mechanism secondary to altered cAMP levels which would be caused by an increased D_1_ receptor stimulation dominating D_2_ receptor stimulation. This theory is sustained by the work of Jäger et al. and Handa et al. who showed that cAMP binds to the GAF-B domain of PDE10A and that this binding activates PDE10A enzymatic activity (Jäger et al. 2012; Handa et al. 2008). Their data are however still controversial since Matthiesen et al. and Russwurm et al. did not observe any changes in PDE10A activity secondary to cAMP alterations (Matthiesen and Nielsen 2009; Russwurm et al. 2015). Further research will be necessary to investigate the exact mechanism of increased PDE10A binding observed in our studies.

In order to clarify whether the D_2_ receptor plays a role in this the chronic response, a subset of the 5-days D-amphetamine treated animals (*n* = 3) was additionally scanned with ^11^C labeled raclopride. If the D_2_ receptor would be involved, changes in D_2_ receptor imaging would have been expected. No significant changes could however be found after the treatment or washout period compared to baseline. These results were further confirmed by D_2_ receptor mRNA quantification which detected no change in D_2_ receptor mRNA levels. These data were also in line with data acquired by Richtand et al., who also observed no change in transcription of the DA receptors after a five-days D-amphetamine treatment (Richtand et al. 1997).

The role of the D_2_ receptor in sensitization can be further questioned based on results obtained by Dlaboga et al. (Dlaboga et al. 2008). In their research, they used quantitative immunoblot analysis to quantify PDE10A expression after treatment with haloperidol, a selective D_2/3_ receptor antagonist. After a 21-day treatment of rats with haloperidol, they could detect a significant increase in PDE10A expression. When the D_2_ receptor on the other hand would be activated by D-amphetamine treatment, a decreased PDE10A binding can be expected. We however discovered that repeated stimulation of the DA neurotransmission by D-amphetamine increases in vivo PDE10A binding. Overall, our results and the above mentioned previous literature, gives us indications that the D_2_ receptor does not play a dominant role in the chronic D-amphetamine response. These data were however later contradicted by Natesan et al. who did not observe any change in PDE10A expression and PET signal after chronic haloperidol treatment (28 days) (Natesan et al. 2014).

In order to further confirm the hypothesis that activation of the D_1_ receptor pathway is responsible for the observed increase in PDE10A binding, we chronically treated another group of rats with a combination of D-amphetamine and SCH23390, a selective inhibitor of the D_1_ receptor (Experiment 6) (Bourne 2001). The dose we used for SCH23390 treatment (1 mg/kg every day) has previously been reported (Hess et al. 1986). Simultaneous activation of the DA neurotransmission, combined with selective blocking of the D_1_ receptor pathway significantly decreased PDE10A availability (Fig. 4). This confirms that after chronic stimulation of the dopaminergic system, D_1_ and not D_2_ receptor activation is likely responsible for the observed increase in PDE10A binding. The importance of the D_1_ receptor after chronic DA stimulation was also suggested by data of Selemon et al. who noticed that D_1_ receptor antagonism can completely reverse the effects of D-amphetamine sensitization (Selemon et al. 2010). Treatment of rats with only SCH23390 for 5 days decreased the PDE10A binding slightly more compared to the rats treated with a combination of D-amphetamine and SCH23390. This difference can be expected since D-amphetamine increases the synaptic DA levels which compete with SCH23390 for D_1_ receptor binding. Considering the higher affinity of SCH23390 to the D_1_ receptor than endogenous DA (Bourne 2001), the relative decrease in PDE10A binding in the SCH23390 treated animals does not differ much from that in the SCH23390/D-amphetamine treated group.

Finally, we further investigated the alteration in [^18^F]JNJ42259152 BP_ND_ observed in D-amphetamine treated animals. Since BP_ND_ equals in vivo ratio of B_max_ over K_D_, the observed increase in [^18^F]JNJ42259152 BP_ND_ could potentially be caused by a change in PDE10A expression or/and a change in affinity of the tracer to PDE10A. In order to differentiate between these two potential mechanisms, we investigated PDE10A mRNA levels in striatum of 5-days D-amphetamine treated animals. PDE10A mRNA levels can be an indication for PDE10A expression, however changes in translation, mobilization of PDE10A and activation of PDE10A could not be excluded. Redistribution of PDE10A secondary to changes in cAMP could also have contributed to the observed change. Several research groups have shown that PDE10A localization can be regulated in response to changes in cAMP levels (Russwurm et al. 2015; Charych et al. 2010). Our data did not show any significant difference in mRNA levels between D-amphetamine treated and saline treated animals. This indicates that the observed changes in [^18^F]JNJ42259152 BP_ND_ might be caused by a change in [^18^F]JNJ42259152 affinity for PDE10A rather than a change in PDE10A expression. Although the differences are small, our data demonstrate changes in PDE10A BP_ND_ independent from PDE10A expression. Since BP_ND_ values are often used in quantification of PDE10A as a measure of expression, it is important to also take affinity effects into account when quantifying PDE10A. Additionally, interactions between DA neurotransmission clearly point out that PDE10A imaging might be biased in patients treated with dopaminergic drugs.

The mechanism by which the affinity increased was not investigated in this study, however this could be a response mechanism to the increased cAMP levels in D-amphetamine treated animals. Although there still is some controversy on the matter, Jäger et al. observed that binding of cAMP to the regulatory GAF domain of PDE10A activates the enzyme (Jäger et al. 2012). A potential mechanism for PDE activation was suggested by Pandit et al. who investigated allosteric regulation of PDE2. In native state, catalytic sites of dimerized PDE2 are packed against each other. Binding of cGMP to the GAF domain of PDE2 rotates the catalytic domain and facilitates the binding of cAMP (Pandit et al. 2009). A similar mechanism for PDE10A can be conceivable since PDE10A also has an allosteric binding site for cAMP. Since [^18^F]JNJ42259152 binds to the same pocket in the catalytic domain as cAMP, activation of PDE10A would not only facilitate cAMP but also [^18^F]JNJ42259152 binding to the catalytic domain. Further investigation of the effect of cAMP on [^18^F]JNJ42259152 binding is currently ongoing to fully understand the mechanism of the alterations observed here.

## Conclusion

In conclusion, we showed that chronic activation of DA neurotransmission increases striatal [^18^F]JNJ42259152 binding to PDE10A. Since many drugs used in neuropsychiatry alter DA neurotransmission, PDE10A binding quantified by PET imaging in CNS disorders may be biased by treatment with dopaminergic drugs Activation of the D_1_ over the D_2_ receptor pathway is responsible for the effects of chronic D-amphetamine exposure.

## Additional files

Additional file 1: **PDE10A binding potential and specific activity.** Correlation analysis of the acquired BP_ND_ values in baseline conditions and the specific activity used for their respective microPET scans. No significant correlation could be found (*p* = 0.3858). (DOCX 30 kb)

Additional file 2: **PDE10A binding potential and supplier of rats.** Overview of baseline BP_ND_ values acquired in Wistar rats supplied by Harlan and Janvier. Baseline BP_ND_ values acquired in rats from Janvier were significantly higher compared to baseline BP_ND_ values acquired in rats from Harlan (Non parametric Mann-Whitney test, *p* = 0.0012). (DOCX 33 kb)

## Abbreviations

AC: adenylyl cyclase
BP_ND_: binding potential value
cAMP: cyclic adenosine monophosphate
cGMP: cyclic guanosine monophosphate
DA: dopamine
i.v.: intravenous
MSN: medium spiny neurons
PDE10A: phospodiesterase 10A
PET: positron emission tomography
PKA: protein kinase A
s.c.: subcutaneous
SCH23390: 8-chloro-3-methyl-5-phenyl-2,3,4,5-tetrahydro-1H-3benzazepin-7-ol
