# Supplementary material for: Striatal phosphodiesterase 10A availability is altered secondary to chronic changes in dopamine neurotransmission
Source: EJNMMI Radiopharm Chem. 2016 Mar 21;1:3. doi: 10.1186/s41181-016-0005-5 (PMC5843803; doi:10.1186/s41181-016-0005-5)
Supplement: Supplementary file 1 — PDE10A binding potential and specific activity. Correlation analysis of the acquired BPND values in baseline conditions and the specific activity used for their respective microPET scans. No significant correlation could be found (p = 0.3858). (DOCX 30 kb) [file 41181_2016_5_MOESM1_ESM.docx]

**PDE10A binding potential and specific activity.** Correlation analysis of the acquired BP_ND_ values in baseline conditions and the specific activity used for their respective microPET scans. No significant correlation could be found (p = 0.3858)
