# Supplementary material for: Striatal phosphodiesterase 10A availability is altered secondary to chronic changes in dopamine neurotransmission
Source: EJNMMI Radiopharm Chem. 2016 Mar 21;1:3. doi: 10.1186/s41181-016-0005-5 (PMC5843803; doi:10.1186/s41181-016-0005-5)
Supplement: Supplementary file 2 — PDE10A binding potential and supplier of rats. Overview of baseline BPND values acquired in Wistar rats supplied by Harlan and Janvier. Baseline BPND values acquired in rats from Janvier were significantly higher compared to baseline BPND values acquired in rats from Harlan (Non parametric Mann-Whitney test, p = 0.0012). (DOCX 33 kb) [file 41181_2016_5_MOESM2_ESM.docx]

**PDE10A binding potential and supplier of rats.** Overview of baseline BP_ND_ values acquired in Wistar rats supplied by Harlan and Janvier. Baseline BP_ND_ values acquired in rats from Janvier were significantly higher compared to baseline BP_ND_ values acquired in rats from Harlan (Non parametric Mann-Whitney test, p = 0.0012).
